# Endoplasmic reticulum calcium stores in dendritic spines

**DOI:** 10.3389/fnana.2014.00064

**Published:** 2014-07-09

**Authors:** Menahem Segal, Eduard Korkotian

**Affiliations:** Department of Neurobiology, The Weizman InstituteRehovot, Israel

**Keywords:** dendritic spines, ryanodine receptors, IP3 receptors, STIM, Orai

## Abstract

Despite decades of research, the role of calcium stores in dendritic spines structure, function and plasticity is still debated. The reasons for this may have to do with the multitude of overlapping calcium handling machineries in the neuron, including stores, voltage and ligand gated channels, pumps and transporters. Also, different cells in the brain are endowed with calcium stores that are activated by different receptor types, and their differential compartmentalization in dendrites, spines and presynaptic terminals complicates their analysis. In the present review we address several key issues, including the role of calcium stores in synaptic plasticity, their role during development, in stress and in neurodegenerative diseases. Apparently, there is increasing evidence for a crucial role of calcium stores, especially of the ryanodine species, in synaptic plasticity and neuronal survival.

## Introduction

Dendritic spines are the locus of most excitatory synapses in the forebrain, and are subject to extensive modifications in the process of growth, maturation and aging (Sala and Segal, 2014). The main route for modifying the structure and functions of dendritic spines is through influx of calcium from the extra synaptic space into the dendritic spine, where an excess calcium concentration triggers cascades of molecular events leading to these changes. While the concentration of free intracellular calcium concentration [Ca^2+^]*_i_* in neurons is very low (in the range of 10–100 nM), about four order of magnitude below the ambient extracellular [Ca^2+^], some intracellular organelles contain fairly high [Ca^2+^]*_i_*, similar to that of [Ca^2+^]o, and are considered to be “calcium stores”. Under certain conditions, the nucleus can contain high concentrations of calcium (Korkotian and Segal, 1996). A more ubiquitous organelle is the mitochondrion, where [Ca^2+^] can reach 100 μM by pumping it in using a specialized uniporter (Rizzuto et al., 2012). Mitochondrial calcium plays an important role in energy metabolism and cell survival, but will not be dealt with herein. The other organelle containing a high [Ca^2+^]*_i_* which is considered to be a “calcium store” is the endoplasmic reticulum (ER), which is ubiquitous in dendrites, and is assumed to extend into spines. The first suggestion for a possible calcium store in dendritic spines, and more specifically in the spine apparatus (SA) is that of Fifková et al. (1983) who conducted one of the earliest electron microscopic (EM) studies to find apparent calcium deposits in the dendritic spines, in close association with the SA. They suggested that the SA is a calcium sequestering organelle, which regulates intraspinal calcium concentration during synaptic activity. A later study using three-dimensional reconstruction of dendritic spines (Spacek and Harris, 1997) revealed a continuum of the smooth ER into the SA, where it forms the typical lamellar structure, which may occasionally reach the postsynaptic density (PSD) at the synapse. Thus, there is a putative structure within the dendritic spine, which may serve a role as a calcium store. The identity and putative functions of this store in dendritic spines is currently subject to extensive analysis.

## The IP3 receptor

The ER calcium stores are activated by two types of receptors, the inositol 1,4,5 trisphosphate receptor (IP3R), and the ryanodine receptor (RyR). The IP3R is of three isoforms, 1–3, and the predominant neuronal one is the IP3R1, whereas the other two are found primarily in non neuronal tissue (Fujino et al., 1995). The RyR, which is activated by low concentrations of ryanodine also has three isoform, RyR1–3, which are found primarily in muscle cells, and are responsible for contraction, but are also found throughout the brain (Galeotti et al., 2008; Kushnir et al., 2010). Both IP3R1 and RyR are distributed across the entire nervous system, with differential distribution in different neuron type and neuronal compartment. EM analysis of the hippocampus indicated that IP3R are present at high concentrations in dendritic shafts and cell bodies, whereas RyR are present primarily in dendritic spines and axons (Sharp et al., 1993). In contrast, GABAergic neurons of the cerebellum contain high concentrations of IP3R, also in dendritic spines. Consequently, IP3Rs were studied extensively in cerebellar purkinje cells (Goto and Mikoshiba, 2011). IP3 stores are important for maintenance of dendritic spine morphology of purkinje cells in the cerebellum (Sugawara et al., 2013). A slow [Ca^2+^]*_i_* surge, resulting from activation of the IP3-receptors has been described in dendritic spines of cerebellar purkinje cells which may have some functional significance in these dendritic spines (Rose and Konnerth, 2001).

Even though the IP3 receptor is not localized in dendritic spines of hippocampal neurons, its involvement in plastic processes in this structure is well documented. IP3Rs are assumed to mediate the action of acetylcholine (ACh) and other neuromodulators, to cause release of calcium from stores and a subsequent change in AMPA and NMDA receptor functions (Raymond and Redman, 2006; Fernández de Sevilla et al., 2008; Fernández de Sevilla and Buño, 2010). IP3Rs probably mediate the effects of brain derived neurotrophic factor (BDNF) on neuronal plasticity in cultured cortical neurons (Nakata and Nakamura, 2007). One possible mode of involvement of ER in calcium release from IP3 stores has been proposed recently (Holbro et al., 2009); synaptic activation of glutamate receptors could evoke a delayed calcium surge in large spines that was blocked by metabotropic glutamate receptor (mGluR) antagonists and heparin (an antagonist of calcium release from IP3 stores). The role of these delayed calcium surges in synaptic plasticity is not entirely clear. A more direct role of IP3Rs in synaptic functions is indicated by Sala et al. (2005), suggesting that activation of mGluR which are located in dendritic spines, recruits IP3Rs to release calcium from stores, so as to activate calcium-gated K currents, which will modulate the efficacy of the transfer of synaptic currents from the synapse on the spine head to the dendritic shaft.

## The ryanodine receptor

The more controversial store is the one associated with the brain RyR (Verkhratsky, 2005; Zalk et al., 2007). There are three isoforms of ryanodine receptors (RyR1–3) that are differentially localized in dendrites and spines of central neurons. Several RyR gene knockdown express specific behavioral phenotypes including an antidepressant effect (Galeotti et al., 2008; Kushnir et al., 2010). Its main attribute is that it is activated by calcium influx into the cell, such that the influx of calcium ions through the plasma membrane is amplified by release of calcium from RyR-associated calcium stores. Caffeine, and low concentration of ryanodine (0.5–1 μM) are agonists for this receptor, and high concentration of ryanodine (100 μM) or cyclopiazonic acid (CPA) are antagonists of the RyR. Initial studies (Mainen et al., 1999; Kovalchuk et al., 2000) were unable to detect an effect of CPA on subthreshold synaptically evoked rise of [Ca^2+^]*i* in CA1 neurons of the hippocampus, a response that was shown to be primarily mediated by activation of the NMDA receptor. In contrast, Emptage et al. (1999) demonstrated that release from stores is responsible for the rise of [Ca^2+^]*i* that is seen following synaptic activation of CA1 neurons of cultured hippocampal slices in that this rise is completely blocked by CPA or ryanodine, an antagonist of the RyR. Interestingly, they did confirm that blockade of calcium-induced release of calcium from stores prolongs the decay of the calcium rise following back propagating action potential, as seen by Mainen et al. (1999) and Sabatini et al. (2002). All three groups found that the size of the calcium transient associated with an action potential is not affected by CPA. The reasons for the difference in the assumed store involvement in the synaptic calcium rise between the Sabatini/Mainen and the Emptage groups is not entirely clear, and it may have to do with the type of preparation, acute slices vs. cultured slice or the intracellular concentration and affinity of calcium sensor used by the three groups (Fluo-4, low concentration, high affinity dye, vs. high concentration, low affinity Oregon Green dye, in the Sabatini/Mainen and Emptage groups, respectively), as well as the sensitivity of the method of recording/imaging.

The issue remained controversial when Brünig et al. (2004) reported that they could not detect any effect of caffeine on spine motility, unlike the effects of NMDA or AMPA receptor activation. Furthermore, Harvey and Svoboda (2007) could not confirm an involvement of RyR in tetanic stimulation-induced spine head expansion, although Harvey and Collingridge (1992) reported that thapsigargin blocks LTP in hippocampal slices. These observations contrast with the effects of caffeine on [Ca^2+^]*i* and spine morphology reported before (Korkotian and Segal, 1999), and the description of the presence of calcium stores in dendritic spines (Harris, 1999). A more recent study supported the link between stores and spines by proposing that the RyR 2 and 3 isoforms mediate the action of BDNF on dendritic spines and on cognitive tasks associated with the hippocampus (Adasme et al., 2011).

A possible molecular and structural substrate associated with the RyR is the SA, which is enriched with synaptopodin (SP), an actin binding protein that was originally detected in the kidney, and later in the brain (Mundel et al., 1997; Deller et al., 2003; Asanuma et al., 2005). Several studies contributed to the initial association of SP with synaptic plasticity. In one, SP expression was found to be enhanced following tetanic stimulation, an effect that is assumed to underlie a transition from short to long-term potentiation (Yamazaki et al., 2001; Fukazawa et al., 2003). It was also demonstrated recently that rats exposed to an acute swim stress expressed a rapid increase in SP-density specifically in the dorsal hippocampus (Vlachos et al., 2008). Thus, dynamic changes in SP accompany plasticity-related stimulation, and are expected to play a role in enhanced memory formation in the behaving animal. It has been proposed that SP is co-localized with the RyR in dendritic spines of rat hippocampus (Vlachos et al., 2009; Segal et al., 2010). Furthermore, a reduction in SP in cultured hippocampal neurons by transfection with RNAi construct led to a marked reduction in plasticity of dendritic spines following several procedures commonly known to generate spine plasticity (ibid). The association of SP with RyR may resolve the inconsistencies among reports on the involvement of RyR in synaptic plasticity, as only about a third of the spines contain the spine apparatus and SP, while the others may not be affected by activators of the RyR (Segal et al., 2010).

**Figure 1:**
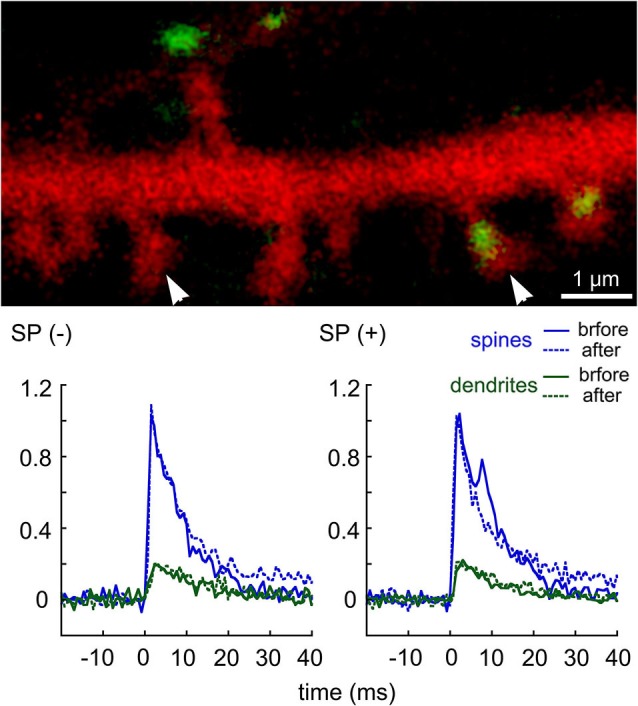
**Transient rise of [Ca^2+^]*_i_* following flash photolysis of caged EGTA in dendritic spines of cultured hippocampal neurons**. DsRed-transfected cells were loaded with a calcium indicator Fluo-4, and with the caged EGTA, the flash was focused on different dendritic spines in the field of view, and the transient elevation of [Ca^2+^]*_i_* was recorded using line scan mode across the spine head and parent dendrite. The culture was then exposed to thapsigargin, and the same spines were imaged again. Following the experiment, cultures were fixed and immunostained for SP. Comparisons were made between SP-containing (right arrowhead) and SP-lacking spines (left arrowhead). For each sample (*n* = 9 spines), the transient rise of [Ca^2+^]*_i_* in both the spine head (blue) and parent dendrite (green) were plotted, before (continuous line) and after thapsigargin (dotted line). A secondary “hump” in the transient rise of [Ca^2+^]*i* was seen only in the SP(+) spines but not in the SP(−) ones. This hump was erased in the presence of thapsigargin (after). Scale in image, 1 μm, ordinate is the averaged transients (df/f). (modified from Korkotian and Segal, 2011).

A more direct indication for a role of RyR in dendritic spine [Ca^2+^]*_i_* variations has been proposed recently by the use of flash photolysis of caged calcium inside dendritic spines (Korkotian and Segal, 2011; Figure 1). A momentary increase in [Ca^2+^]*_i_* in dendritic spines by photolysis of caged calcium is followed by an exponential decay back to baseline level. However, in spines that are endowed with a SP puncta, an additional slow non-exponential component of elevated [Ca^2+^]*_i_* was seen, and this was abrogated by RyR antagonists, indicating that a rise in free [Ca^2+^]*_i_* is followed by a further increase, caused by its release from local stores.

## Calcium stores and plasticity

The involvement of calcium stores in generation and maintenance of long term potentiation has been studied extensively over the past two decades (Meldolesi, 2001; Shimuta et al., 2001; Fitzjohn and Collingridge, 2002; Bardo et al., 2006; Baker et al., 2013). Several compounds, known to produce long term enhancement of synaptic reactivity, have been suggested to act by releasing of calcium from stores (Auerbach and Segal, 1994; Welsby et al., 2006). Furthermore, direct release of calcium from stores, using caffeine, produced a calcium store-dependent, NMDA-independent LTP (Martin and Buno, 2003). These authors suggest that the LTP is mediated presynaptically, to affect release of glutamate from terminals. Likewise, a brief exposure to caffeine, acting via a RyR, primed the conversion of short term to long term potentiation (Sajikumar et al., 2009). These and similar studies where tetanus-induced LTP could be blocked by drugs which interfere with the release of calcium from stores (Harvey and Collingridge, 1992) demonstrate the importance of calcium stores in the generation and maintenance of LTP. An interesting intra-hippocampal differential distribution of RyRs across the septo-temporal axis has been demonstrated (Grigoryan et al., 2012), which affects the ability of ryanodine and caffeine to enhance LTP in CA1 region of the hippocampal slice. This indicates that the activation of RyR in relation to neuronal plasticity is likely to be region-specific. Furthermore, the involvement of the stores can be exerted through the regulation of delivery of glutamate receptors to the synaptic site (Korkotian and Segal, 2006, 2007), or through regulation of one of several calcium-dependent processes in the dendrite, the spine or the synapse.

## Loading of calcium into stores

**Figure 2:**
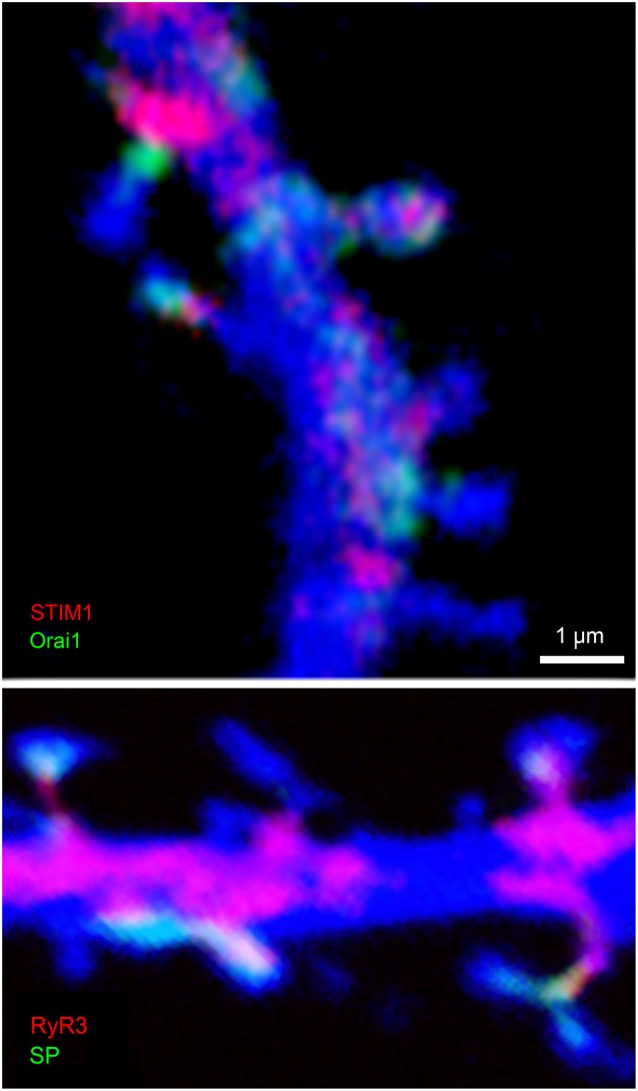
**Colocalization of STIM1 and Orai1 (top) and RyR3 and SP (bottom) in cultured hippocampal neurons**. Cells were transfected with DsRed to image morphology (blue) and subsequently immunostained for STIM1 (red, top) and Orai1(green), or RyR (red) and SP (green, bottom). Scale 1 μm for both images. Colocalization of STIM and Orai are obvious in some but not all spines, such that either both or none were immunostained in the dendritic spines. Similarly, RyR and SP were colocalized in some but not all dendritic spines.

RyRs are unique in that they are activated by an influx of calcium to induce release of calcium from the stores. That is, if calcium ions enter the cell via voltage or ligand gated channels, the RyR store cause further release of calcium ions, so as to amplify the action of the influxed ions. However, the influx of calcium through ion and ligand gated channels is only one of two main mechanisms that feed calcium into the stores. The other one is independent of the synapse-related ion channels, and is unique in that it senses a reduction in calcium concentrations in the store, to activate influx of calcium directly through the membrane, irrespective of afferent activity. This process is called store-operated calcium entry (SOCE) and has been studied extensively in non-neuronal tissue, with only few studies examining its role in central neurons. The reduction of [Ca^2+^]*_i_* in the stores is sensed by stromal interacting protein (STIM), which is localized to the cytoplasm. Upon demand (i.e., low [Ca^2+^]*_i_*), STIM moves to the plasma membrane where it binds to Orai, a calcium channel that is voltage and extracellular ligand-independent. Both STIM and Orai are found in some dendritic spines (Figure 2). Following binding to STIM, Orai allows influx of calcium ions into the cytoplasm, where they are pumped into the ER stores. The malfunctioning of peripheral STIM/Orai complex has been associated with severe immunodeficiency syndrome (Feske et al., 2010). In the brain there are two species of STIM, STIM1, which has been shown recently to link to mGluRs and play a critical role in cerebellar neurons (Hartmann et al., 2014), and STIM2 (Sun et al., 2014) which appears to regulate influx of calcium in forebrain neurons, and be related to Alzheimer’s disease (AD) (below). STIM and Orai have been identified in several neuronal compartments (Korkotian et al., 2014), and they are assumed to play an important role in the formation and maturation of dendritic spines.

There is a long standing issue concerning the development of spines; what is formed first, spines which grow in search for a presynaptic partner, or synapses that are made first on the dendritic shaft, followed by extension and formation of spines. If indeed Orai allows influx of calcium that is independent of synaptic activity, it can be considered to have a pivotal role in spine formation, and help form spines even in the absence of a synapse. Consequently, spines are not formed by an extrinsic factor, e.g., presynaptic activity, but by an intrinsic need to fill up stores, which allows a local rise of [Ca^2+^]*_i_*, sufficient to activate actin polymerization and formation of spines, which then hunt for a presynaptic partner, or collapse, in its absence. In any case, the presence of Orai in dendritic spines is an additional evidence for the important role of calcium stores in dendritic spine formation and plasticity.

## Calcium stores and stress

Exposure to stress can cause lasting effects in specific regions in the brain including the hippocampus. Following stressful experience, CA1 region of the dorsal hippocampus shows a lower ability to express LTP, and a larger long term depression (LTD) (Maggio and Segal, 2007). Surprisingly, CA1 of ventral hippocampus (VH) expresses larger LTP following the same stress. In previous studies we ascribed this metaplastic properties of VH to the recruitment of calcium stores (Grigoryan et al., 2012). In more recent studies, stress has been shown to cause an increase in beta adrenergic receptor activation of protein kinase A (PKA), which in turn regulates release of calcium from stores (Grigoryan and Segal, 2013). Dysregulation of RyR activation by the enhanced PKA results in release of calcium from stores, which perhaps can enhance ability to express LTP, but may also have long term detrimental consequences to cell function and survival (Stutzmann and Mattson, 2011; Liu et al., 2012). While these studies do not address directly the role of RyR in dendritic spines, there is accumulating evidence that chronic stress severely reduces dendritic spine density as well as ability to express LTP, which may be directly related to the deterioration of mechanisms for regulation of release of calcium from stores (Andres et al., 2013).

## Calcium stores in neurodegenerative diseases

Several studies suggest that synapse and dendritic spines dysfunctions are preceding and contributing to the eventual neuronal death and neurodegeneration in AD (Youn et al., 2007; Yu et al., 2012). Strikingly, recent work by Stutzmann and Mattson (2011) associates AD neuropathology with an over-expression of RyRs in dendrites and particularly in spines. Furthermore, caffeine caused a large facilitation of reactivity to tetanic stimulation in neurons from young 3xTg mouse hippocampus but not from controls (Grigoryan et al., 2014). This supersensitivity to activation of the RyR was noticed before the emergence of neuropathology in these mice, indicating that activation of RyR stores may lead rather than lag behind the pathology of AD. These results indicate that the association of RyR with dendritic spines, and their role in release of calcium from stores in relation to synaptic plasticity may be more important than originally suggested, especially in the development of AD.

This is also supported by post-mortem tissue sample analysis from patients with AD that consistently showed prominent synapse and dendritic spine loss in the hippocampus and throughout the cortex, the principal areas affected by AD-related pathology (DeKosky and Scheff, 1990; Knafo et al., 2009).

Indeed the number of synapses and spines elimination is often greater than the expected level for the amount of lost neurons and better correlates with the cognitive decline, suggesting that the primary synaptic failure is a prominent pathogenic cause of AD (Walsh and Selkoe, 2004; Verpelli and Sala, 2012).

The identification of specific genes associated with the pathogenesis of AD will lead to a better understanding of the molecular mechanisms underlying synapse and spine alterations. Mutations in three major genes implicated in beta amyloid (Aβ) metabolism, amyloid precursor protein (APP) and presenilin1 (PS1) have been associated to familial AD with an autosomal dominant form of inheritance and early onset of the disease (Bertram and Tanzi, 2008; Bertram et al., 2010). These mutations cause a well-documented increase in production of Aβ oligomers that at the end are responsible for inducing spine alterations, and reduce spine density (Shankar et al., 2007).

Mouse models of AD show behavioral deficits in reference and working memory but also dendritic and synaptic alterations, similar to what can be described in human patients. For example the human APP Tg2576 transgene mouse model shows a reduced spine density in hippocampal neurons from CA1 and dentate gyrus, even before the development of amyloid plaques, suggesting that soluble pathological Aβ oligomers initiate synapses alterations before neurodegeneration (Middei et al., 2008). Similarly, APP/PS1 transgenic mice showed alterations in spine density and morphology in dendrites in proximity of plaques but also a significant decrease in the frequency of large spines in neurons of plaque-free regions (Knafo et al., 2009).

Amyloid peptides have been shown to cause calcium rise, and neuronal degeneration. Paula-Lima et al. (2011) proposed that the amyloid oligomers stimulate RyR mediated calcium release, to induce mitochondrial fragmentation and loss of calcium regulation, ending in cell death. On the other hand, Zeiger et al. (2013) suggest that calcium influx through SOCE actually reduces formation of amyloid beta peptide, the evil molecule in AD. Thus, whether activation of SOCE is beneficial or detrimental to cell function in an AD environment remains to be elucidated. A more recent study, using Presenilin-mutant, familial AD-like mouse model, found that suppression of IP3Rs alleviates the AD symptoms. This study links calcium stores to AD, and proposes novel strategies for the treatment of the disease.

Many other genes have been identified as high risk factors to develop AD in the elderly. Among these genes apolipoprotein E (APOE) is the most important and studied risk factor. ApoE has three major isoforms, apoE2, E3 and E4 and apoE4 is associated with high risk of developing the disease, while the other ApoE seem to be neuroprotective. Transgenic mice for the human ApoE isoforms showed that indeed ApoE4 transgene has reduced spine density in the dentate gyrus and in the cortex in an age-dependent manner, while mice expressing human ApoE2 and ApoE3 have normal numbers of spines. One study also showed that in the human brain there is an inverse correlation between ApoE4 expression and spine density in dentate gyrus neurons (Dumanis et al., 2009). Interestingly the transgene expression of ApoE2 in two different mouse models of AD (Tg2576 and PDAPP mice) can rescue spine density to normal levels (Lanz et al., 2003). Thus all the mouse models of AD show alteration of spine morphology that correlates well with synaptic and behavioral dysfunctions. Consequently, measuring spine morphology is a good readout of the progression and severity of the pathology.

Which are the molecular mechanisms causing spine degeneration in AD? Recent results suggest that some signalling pathways regulating synaptic plasticity are involved. For example cofilin and drebrin, the actin-binding proteins with opposite effects on actin dynamics (see above), are both affected in AD. Cofilin phosphorylation and activity is up-regulated by Aβ 1–42 peptide in a concentration-dependent manner thus causing alteration in actin polymerization (Knobloch and Mansuy, 2008). In striking contrast, drebrin, the postsynaptic protein that binds and stabilizes actin in spines, is severely reduced in the brains of patients with AD and in transgenic animal models of the disease (Counts et al., 2012). Thus, a major initiating factor is probably associated with the malfunction of the RyR, causing a change in basal [Ca^2+^]*_i_*, and consequently changes in calcium associated processes responsible for the spine cytoskeleton. Current studies (Shilling et al., 2014; Sun et al., 2014) attempt to ameliorate the AD-associated morphological deterioration by addressing specifically calcium stores.

## Conclusions

Recent accumulating evidence indicates that calcium stores of the RyR type have a pivotal role in dendritic spine development, plasticity and longevity. Disruption of this role of RyR in any stage of life will retard the functionality of the spine, the parent dendrite and the neuron of origin, leading to mental deterioration and AD. Recent attempts to ameliorate AD development focus on the RyR, and the regulation of [Ca^2+^]*_i_* in the affected neurons.

## Author contributions

Both Menahem Segal and Eduard Korkotian wrote the manuscript.

## Conflict of interest statement

The authors declare that the research was conducted in the absence of any commercial or financial relationships that could be construed as a potential conflict of interest.

## References

[B1] Adasme T. Haeger P. Paula-Lima A. C. Espinoza I. Casas-Alarcón M. M. Carrasco M. A. (2011). Involvement of ryanodine receptors in neurotrophin-induced hippocampal synaptic plasticity and spatial memory formation. Proc. Natl. Acad. Sci. U S A 108, 3029–3034 10.1073/pnas.1013580108 21282625 PMC3041089

[B2] Andres A. L. Regev L. Phi L. Seese R. R. Chen Y. Gall C. M. (2013). NMDA receptor activation and calpain contribute to disruption of dendritic spines by the stress neuropeptide CRH. J. Neurosci. 33, 16945–16960 10.1523/JNEUROSCI.1445-13.2013 24155300 PMC3807024

[B3] Asanuma K. Kim K. Oh J. Giardino L. Chabanis S. Faul C. (2005). Synaptopodin regulates the actin-bundling activity of alpha-actinin in an isoform-specific manner. J. Clin. Invest. 115, 1188–1198 10.1172/jci200523371 15841212 PMC1070637

[B4] Auerbach J. M. Segal M. (1994). A novel cholinergic induction of long-term potentiation in rat hippocampus. J. Neurophysiol. 72, 2034–2040 7823117 10.1152/jn.1994.72.4.2034

[B5] Baker K. D. Edwards T. M. Rickard N. S. (2013). The role of intracellular calcium stores in synaptic plasticity and memory consolidation. Neurosci. Biobehav. Rev. 37, 1211–1239 10.1016/j.neubiorev.2013.04.011 23639769

[B6] Bardo S. Cavazzini M. G. Emptage N. (2006). The role of the endoplasmic reticulum Ca2+ store in the plasticity of central neurons. Trends Pharmacol. Sci. 27, 78–84 10.1016/j.tips.2005.12.008 16412523

[B7] Bertram L. Lill C. M. Tanzi R. E. (2010). The genetics of Alzheimer disease: back to the future. Neuron 68, 270–281 10.1016/j.neuron.2010.10.013 20955934

[B8] Bertram L. Tanzi R. E. (2008). Thirty years of Alzheimer’s disease genetics: the implications of systematic meta-analyses. Nat. Rev. Neurosci. 9, 768–778 10.1038/nrn2494 18802446

[B9] Brünig I. Kaech S. Brinkhaus H. Oertner T. G. Matus A. (2004). Influx of extracellular calcium regulates actin-dependent morphological plasticity in dendritic spines. Neuropharmacology 47, 669–676 10.1016/j.neuropharm.2004.07.038 15458838

[B10] Counts S. E. He B. Nadeem M. Wuu J. Scheff S. W. Mufson E. J. (2012). Hippocampal drebrin loss in mild cognitive impairment. Neurodegener. Dis. 10, 216–219 10.1159/000333122 22310934 PMC3363353

[B11] DeKosky S. T. Scheff S. W. (1990). Synapse loss in frontal cortex biopsies in Alzheimer’s disease: correlation with cognitive severity. Ann. Neurol. 27, 457–464 10.1002/ana.410270502 2360787

[B12] Deller T. Korte M. Chabanis S. Drakew A. Schwegler H. Stefani G. G. (2003). Synaptopodin-deficient mice lack a spine apparatus and show deficits in synaptic plasticity. Proc. Natl. Acad. Sci. U S A 100, 10494–10499 10.1073/pnas.1832384100 12928494 PMC193589

[B13] Dumanis S. B. Tesoriero J. A. Babus L. W. Nguyen M. T. Trotter J. H. Ladu M. J. (2009). ApoE4 decreases spine density and dendritic complexity in cortical neurons in vivo. J. Neurosci. 29, 15317–15322 10.1523/JNEUROSCI.4026-09.2009 19955384 PMC2846754

[B14] Emptage N. Bliss T. V. Fine A. (1999). Single synaptic events evoke NMDA receptor-mediated release of calcium from internal stores in hippocampal dendritic spines. Neuron 22, 115–124 10.1016/s0896-6273(00)80683-2 10027294

[B15] Fernández de Sevilla D. Buño W. (2010). The muscarinic long-term enhancement of NMDA and AMPA receptor-mediated transmission at Schaffer collateral synapses develop through different intracellular mechanisms. J. Neurosci. 30, 11032–11042 10.1523/JNEUROSCI.1848-10.2010 20720110 PMC6633467

[B16] Fernández de Sevilla D. Núñez A. Borde M. Malinow R. Buño W. (2008). Cholinergic-mediated IP3-receptor activation induces long-lasting synaptic enhancement in CA1 pyramidal neurons. J. Neurosci. 28, 1469–1478 10.1523/JNEUROSCI.2723-07.2008 18256268 PMC6671582

[B17] Feske S. Picard C. Fischer A. (2010). Immunodeficiency due to mutations in ORAI1 and STIM1. Clin. Immunol. 135, 169–182 10.1016/j.clim.2010.01.011 20189884 PMC2856745

[B18] Fifková E. Markham J. A. Delay R. J. (1983). Calcium in the spine apparatus of dendritic spines in the dentate molecular layer. Brain Res. 266, 163–168 10.1016/0006-8993(83)91322-7 6189559

[B19] Fitzjohn S. M. Collingridge G. L. (2002). Calcium stores and synaptic plasticity. Cell Calcium 32, 405–411 10.1016/s0143416002001999 12543099

[B20] Fujino I. Yamada N. Miyawaki A. Hasegawa M. Furuichi T. Mikoshiba K. (1995). Differential expression of type 2 and type 3 inositol 1,4,5-trisphosphate receptor mRNAs in various mouse tissues: in situ hybridization study. Cell Tissue Res. 280, 201–210 10.1007/bf00307790 7781020

[B21] Fukazawa Y. Saitoh Y. Ozawa F. Ohta Y. Mizuno K. Inokuchi K. (2003). Hippocampal LTP is accompanied by enhanced F-actin content within the dendritic spine that is essential for late LTP maintenance in vivo. Neuron 38, 447–460 10.1016/s0896-6273(03)00206-x 12741991

[B22] Galeotti N. Vivoli E. Bartolini A. Ghelardini C. (2008). A gene-specific cerebral types 1, 2 and 3 RyR protein knockdown induces an antidepressant-like effect in mice. J. Neurochem. 106, 2385–2394 10.1111/j.1471-4159.2008.05581.x 18643873

[B23] Goto J. Mikoshiba K. (2011). Inositol 1,4,5-trisphosphate receptor-mediated calcium release in Purkinje cells: from molecular mechanism to behavior. Cerebellum 10, 820–833 10.1007/s12311-011-0270-5 21701896

[B24] Grigoryan G. Biella G. Albani D. Forloni G. Segal M. (2014). Stress impairs synaptic plasticity in triple-transgenic Alzheimer’s disease mice: rescue by ryanodine. Neurodegener. Dis. 13, 135–138 10.1159/000354231 24008840

[B25] Grigoryan G. Korkotian E. Segal M. (2012). Selective facilitation of LTP in the ventral hippocampus by calcium stores. Hippocampus 22, 1635–1644 10.1002/hipo.22000 22271636

[B26] Grigoryan G. Segal M. (2013). Prenatal stress alters noradrenergic modulation of LTP in hippocampal slices. J. Neurophysiol. 110, 279–285 10.1152/jn.00834.2012 23615548

[B27] Harris K. M. (1999). Calcium from internal stores modifies dendritic spine shape. Proc. Natl. Acad. Sci. U S A 96, 12213–12215 10.1073/pnas.96.22.12213 10535897 PMC34250

[B28] Hartmann J. Karl R. M. Alexander R. P. Adelsberger H. Brill M. S. Rühlmann C. (2014). STIM1 controls neuronal Ca(2+) signaling, mGluR1-dependent synaptic transmission and cerebellar motor behavior. Neuron 82, 635–644 10.1016/j.neuron.2014.03.027 24811382

[B29] Harvey J. Collingridge G. L. (1992). Thapsigargin blocks the induction of long-term potentiation in rat hippocampal slices. Neurosci. Lett. 139, 197–200 10.1016/0304-3940(92)90551-h 1319014

[B30] Harvey C. D. Svoboda K. (2007). Locally dynamic synaptic learning rules in pyramidal neuron dendrites. Nature 450, 1195–1200 10.1038/nature06416 18097401 PMC3425382

[B31] Holbro N. Grunditz A. Oertner T. G. (2009). Differential distribution of endoplasmic reticulum controls metabotropic signaling and plasticity at hippocampal synapses. Proc. Natl. Acad. Sci. U S A 106, 15055–15060 10.1073/pnas.0905110106 19706463 PMC2736455

[B32] Knafo S. Alonso-Nanclares L. Gonzalez-Soriano J. Merino-Serrais P. Fernaud-Espinosa I. Ferrer I. (2009). Widespread changes in dendritic spines in a model of Alzheimer’s disease. Cereb. Cortex 19, 586–592 10.1093/cercor/bhn111 18632740

[B33] Knobloch M. Mansuy I. M. (2008). Dendritic spine loss and synaptic alterations in Alzheimer’s disease. Mol. Neurobiol. 37, 73–82 10.1007/s12035-008-8018-z 18438727

[B34] Korkotian E. Segal M. (1999). Release of calcium from stores alters the morphology of dendritic spines in cultured hippocampal neurons. Proc. Nat. Acad. Sci. U S A 96, 12068–12072 10.1073/pnas.96.21.12068 10518577 PMC18413

[B35] Korkotian E. Segal M. (2007). Morphological constraints on calcium dependent glutamate receptor trafficking into individual dendritic spine. Cell Calcium 42, 41–57 10.1016/j.ceca.2006.11.006 17187855

[B36] Korkotian E. Segal M. (2011). Synaptopodin regulates release of calcium from stores in dendritic spines of cultured hippocampal neurons. J. Physiol. 589, 5987–5995 10.1113/jphysiol.2011.217315 22025667 PMC3286680

[B37] Korkotian E. Frotscher M. Segal M. (2014). Synaptopodin regulates spine plasticity: mediation by calcium stores. J. Neurosci. (in press). 10.1523/JNEUROSCI.0381-14.2014 PMC6608418 25164660

[B38] Korkotian E. Segal M. (1996). Lasting effects of glutamate on nuclear calcium concentration in cultured rat hippocampal neurons: regulation by calcium stores. J. Physiol. 496, 39–48 8910194 10.1113/jphysiol.1996.sp021663 PMC1160822

[B39] Korkotian E. Segal M. (2006). Spatially confined diffusion of calcium in dendrites of hippocampal neurons revealed by flash photolysis of caged calcium. Cell Calcium 40, 441–449 10.1016/j.ceca.2006.08.008 17064764

[B40] Kovalchuk Y. Eilers J. Lisman J. Konnerth A. (2000). NMDA receptor-mediated subthreshold Ca(2+) signals in spines of hippocampal neurons. J. Neurosci. 20, 1791–1799 10684880 10.1523/JNEUROSCI.20-05-01791.2000 PMC6772937

[B41] Kushnir A. Betzenhauser M. J. Marks A. R. (2010). Ryanodine receptor studies using genetically engineered mice. FEBS Lett. 584, 1956–1965 10.1016/j.febslet.2010.03.005 20214899 PMC3690514

[B42] Lanz T. A. Carter D. B. Merchant K. M. (2003). Dendritic spine loss in the hippocampus of young PDAPP and Tg2576 mice and its prevention by the ApoE2 genotype. Neurobiol. Dis. 13, 246–253 10.1016/s0969-9961(03)00079-2 12901839

[B43] Liu X. Betzenhauser M. J. Reiken S. Meli A. C. Xie W. Chen B. X. (2012). Role of leaky neuronal ryanodine receptors in stress-induced cognitive dysfunction. Cell 150, 1055–1067 10.1016/j.cell.2012.06.052 22939628 PMC3690518

[B44] Maggio N. Segal M. (2007). Striking variations in corticosteroid modulation of long-term potentiation along the septotemporal axis of the hippocampus. J. Neurosci. 27, 5757–5765 10.1523/jneurosci.0155-07.2007 17522319 PMC6672761

[B45] Mainen Z. F. Malinow R. Svoboda K. (1999). Synaptic calcium transients in single spines indicate that NMDA receptors are not saturated. Nature 399, 151–155 10.1038/20187 10335844

[B46] Martin E. D. Buno W. (2003). Caffeine-mediated presynaptic long term potentiation in hippocampal CA1 pyramidal neurons. J. Neurophysiol. 89, 3029–3038 10.1152/jn.00601.2002 12783948

[B47] Meldolesi J. (2001). Rapidly exchanging Ca2+ stores in neurons: molecular, structural and functional properties. Prog. Neurobiol. 65, 309–338 10.1016/s0301-0082(01)00004-1 11473791

[B48] Middei S. Restivo L. Caprioli A. Aceti M. Ammassari-Teule M. (2008). Region-specific changes in the microanatomy of single dendritic spines over time might account for selective memory alterations in ageing hAPPsweTg2576 mice, a mouse model for Alzheimer disease. Neurobiol. Learn. Mem. 90, 467–471 10.1016/j.nlm.2008.04.008 18515161

[B49] Mundel P. Heid H. W. Mundel T. M. Kruger M. Reiser J. Kriz W. (1997). Synaptopodin: an actin-associated protein in telencephalic dendrites and renal podocytes. J. Cell Biol. 139, 193–204 10.1083/jcb.139.1.193 9314539 PMC2139823

[B50] Nakata H. Nakamura S. (2007). Brain-derived neurotrophic factor regulates AMPA receptor trafficking to post-synaptic densities via IP3R and TRPC calcium signaling. FEBS Lett. 581, 2047–2054 10.1016/j.febslet.2007.04.041 17482902

[B51] Paula-Lima A. C. Adasme T. SanMartín C. Sebollela A. Hetz C. Carrasco M. A. (2011). Amyloid β-peptide oligomers stimulate RyR-mediated Ca2+ release inducing mitochondrial fragmentation in hippocampal neurons and prevent RyR-mediated dendritic spine remodeling produced by BDNF. Antioxid. Redox Signal. 14, 1209–1223 10.1089/ars.2010.3287 20712397

[B52] Raymond C. R. Redman S. J. (2006). Spatial segregation of neuronal calcium signals encodes different forms of LTP in rat hippocampus. J. Physiol. 570, 97–111 10.1113/jphysiol.2005.098947 16284072 PMC1464297

[B53] Rizzuto R. De Stefani D. Raffaello A. Mammucari C. (2012). Mitochondria as sensors and regulators of calcium signaling. Nat. Rev. Mol. Cell Biol. 13, 566–578 10.1038/nrm3412 22850819

[B54] Rose C. R. Konnerth A. (2001). Stores not just for storage. Intracellular calcium release and synaptic plasticity. Neuron 31, 519–522 10.1016/S0896-6273(01)00402-0 11545711

[B55] Sabatini B. L. Oertner T. G. Svoboda K. (2002). The life cycle of Ca(2+) ions in dendritic spines. Neuron 33, 439–452 10.1016/s0896-6273(02)00573-1 11832230

[B56] Sajikumar S. Li Q. Abraham W. C. Xiao Z. C. (2009). Priming of short term potentiation and synaptic tagging/capture mechanisms by ryanodine receptor activation in rat hippocampal CA1. Learn. Mem. 16, 178–186 10.1101/lm.1255909 19223601

[B57] Sala C. Roussignol G. Meldolesi J. Fagni L. (2005). Key role of the postsynaptic density scaffold proteins Shank and Homer in the functional architecture of Ca2+ homeostasis at dendritic spines in hippocampal neurons. J. Neurosci. 25, 4587–4592 10.1523/jneurosci.4822-04.2005 15872106 PMC6725036

[B58] Sala C. Segal M. (2014). Dendritic spines: the locus of structural and functional plasticity. Physiol. Rev. 94, 141–188 10.1152/physrev.00012.2013 24382885

[B59] Segal M. Vlachos A. Korkotian E. (2010). The spine apparatus, synaptopodin and dendritic spine plasticity. Neuroscientist 16, 125–131 10.1177/1073858409355829 20400711

[B60] Shankar G. M. Bloodgood B. L. Townsend M. Walsh D. M. Selkoe D. J. Sabatini B. L. (2007). Natural oligomers of the Alzheimer amyloid-beta protein induce reversible synapse loss by modulating an NMDA-type glutamate receptor-dependent signaling pathway. J. Neurosci. 27, 2866–2875 10.1523/jneurosci.4970-06.2007 17360908 PMC6672572

[B61] Sharp A. H. McPherson P. S. Dawson T. M. Aoki C. Campbell K. P. Snyder S. H. (1993). Differential immunohistochemical localization of inositol 1,4,5-trisphosphate- and ryanodine-sensitive Ca2+ release channels in rat brain. J. Neurosci. 13, 3051–3063 8392539 10.1523/JNEUROSCI.13-07-03051.1993 PMC6576698

[B63] Shilling D. Müller M. Takano H. Mak D. O. Abel T. Coulter D. A. (2014). Suppression of InsP3 receptor-mediated Ca2+ signaling alleviates mutant presenilin-linked familial Alzheimer’s disease pathogenesis. J. Neurosci. 34, 6910–6923 10.1523/JNEUROSCI.5441-13.2014 24828645 PMC4019804

[B64] Shimuta M. Yoshikawa M. Fukaya M. Watanabe M. Takeshima H. Manabe T. (2001). Postsynaptic modulation of AMPA receptor mediated synaptic responses and LTP by the type 3 Ryanodine receptor. Mol. Cell. Neurosci. 17, 921–930 10.1006/mcne.2001.0981 11358488

[B65] Spacek J. Harris K. M. (1997). Three-dimensional organization of smooth endoplasmic reticulum in hippocampal CA1 dendrites and dendritic spines of the immature and mature rat. J. Neurosci. 17, 190–203 8987748 10.1523/JNEUROSCI.17-01-00190.1997 PMC6793680

[B66] Stutzmann G. E. Mattson M. P. (2011). Endoplasmic reticulum Ca(2+) handling in excitable cells in health and disease. Pharmacol. Rev. 63, 700–727 10.1124/pr.110.003814 21737534 PMC3141879

[B67] Sugawara T. Hisatsune C. Le T. D. Hashikawa T. Hirono M. Hattori M. (2013). Type 1 inositol trisphosphate receptor regulates cerebellar circuits by maintaining the spine morphology of purkinje cells in adult mice. J. Neurosci. 33, 12186–12196 10.1523/JNEUROSCI.0545-13.2013 23884927 PMC6618669

[B68] Sun S. Zhang H. Liu J. Popugaeva E. Xu N. J. Feske S. (2014). Reduced synaptic STIM2 expression and impaired store-operated calcium entry cause destabilization of mature spines in mutant presenilin mice. Neuron 82, 79–93 10.1016/j.neuron.2014.02.019 24698269 PMC4007018

[B69] Verkhratsky A. (2005). Physiology and pathophysiology of the calcium store in the endoplasmic reticulum of neurons. Physiol. Rev. 85, 201–279 10.1152/physrev.00004.2004 15618481

[B70] Verpelli C. Sala C. (2012). Molecular and synaptic defects in intellectual disability syndromes. Curr. Opin. Neurobiol. 22, 530–536 10.1016/j.conb.2011.09.007 22000839

[B71] Vlachos A. Korkotian E. Schonfeld E. Copanaki E. Deller T. Segal M. (2009). Synaptopodin regulates plasticity of dendritic spines in hippocampal neurons. J. Neurosci. 29, 1017–1033 10.1523/JNEUROSCI.5528-08.2009 19176811 PMC6665122

[B72] Vlachos A. Maggio N. Segal M. (2008). Lack of correlation between synaptopodin expression and the ability to induce LTP in the rat dorsal and ventral hippocampus. Hippocampus 18, 1–4 10.1002/hipo.20373 17910066

[B73] Walsh D. M. Selkoe D. J. (2004). Deciphering the molecular basis of memory failure in Alzheimer’s disease. Neuron 44, 181–193 10.1016/j.neuron.2004.09.010 15450169

[B74] Welsby P. Rowan M. Anwyl R. (2006). Nicotinic receptor-mediated enhancement of long term potentiation involves activation of metabotropic glutamate receptors and ryanodine-sensitive calcium stores in the dentate gyrus. Eur. J. Neurosci. 24, 3109–3118 10.1111/j.1460-9568.2006.05187.x 17156372

[B75] Yamazaki M. Matsuo R. Fukazawa Y. Ozawa F. Inokuchi K. (2001). Regulated expression of an actin-associated protein, synaptopodin, during long-term potentiation. J. Neurochem. 79, 192–199 10.1046/j.1471-4159.2001.00552.x 11595771

[B76] Youn H. Jeoung M. Koo Y. Ji H. Markesbery W. R. Ji I. (2007). Kalirin is under-expressed in Alzheimer’s disease hippocampus. J. Alzheimers Dis. 11, 385–397 17851188 10.3233/jad-2007-11314

[B77] Yu W. Polepalli J. Wagh D. Rajadas J. Malenka R. Lu B. (2012). A critical role for the PAR-1/MARK-tau axis in mediating the toxic effects of Aβ on synapses and dendritic spines. Hum. Mol. Genet. 21, 1384–1390 10.1093/hmg/ddr576 22156579 PMC3284124

[B78] Zalk R. Lehnart S. E. Marks A. R. (2007). Modulation of the ryanodine receptor and intracellular calcium. Annu. Rev. Biochem. 76, 367–385 10.1146/annurev.biochem.76.053105.094237 17506640

[B79] Zeiger W. Vetrivel K. S. Buggia-Prévot V. Nguyen P. D. Wagner S. L. Villereal M. L. (2013). Ca2+ influx through store-operated Ca2+ channels reduces Alzheimer disease β-amyloid peptide secretion. J. Biol. Chem. 288, 26955–26966 10.1074/jbc.M113.473355 23902769 PMC3772244

